# Financing of U.S. Biomedical Research and New Drug Approvals across Therapeutic Areas

**DOI:** 10.1371/journal.pone.0007015

**Published:** 2009-09-11

**Authors:** E. Ray Dorsey, Joel P. Thompson, Melisa Carrasco, Jason de Roulet, Philip Vitticore, Sean Nicholson, S. Claiborne Johnston, Robert G. Holloway, Hamilton Moses

**Affiliations:** 1 Department of Neurology, University of Rochester Medical Center, Rochester, New York, United States of America; 2 School of Medicine & Biomedical Sciences, University at Buffalo, The State University of New York, Buffalo, New York, United States of America; 3 School of Medicine and Dentistry, University of Rochester, Rochester, New York, United States of America; 4 University Hospitals, Case Medical Center, Cleveland, Ohio, United States of America; 5 Department of Policy Analysis and Management, Cornell University, Ithaca, New York, United States of America; 6 Department of Neurology, University of California San Francisco, San Francisco, California, United States of America; 7 The Alerion Institute, North Garden, Virginia, United States of America; 8 Johns Hopkins University School of Medicine, Baltimore, Maryland, United States of America; Cedars-Sinai Medical Center and University of California Los Angeles, United States of America

## Abstract

**Background:**

We estimated U.S. biomedical research funding across therapeutic areas, determined the association with disease burden, and evaluated new drug approvals that resulted from this investment.

**Methodology/Principal Findings:**

We calculated funding from 1995 to 2005 and totaled Food and Drug Administration approvals in eight therapeutic areas (cardiovascular, endocrine, gastrointestinal, genitourinary, HIV/AIDS, infectious disease excluding HIV, oncology, and respiratory) primarily using public data. We then calculated correlations between funding, published estimates of disease burden, and drug approvals.

Financial support for biomedical research from 1995 to 2005 increased across all therapeutic areas between 43% and 369%. Industry was the principal funder of all areas except HIV/AIDS, infectious disease, and oncology, which were chiefly sponsored by the National Institutes of Health (NIH). Total (ρ = 0.70; P = .03) and industry funding (ρ = 0.69; P = .04) were correlated with projected disease burden in high income countries while NIH support (ρ = 0.80; P = .01) was correlated with projected disease burden globally. From 1995 to 2005 the number of new approvals was flat or declined across therapeutic areas, and over an 8-year lag period, neither total nor industry funding was correlated with future approvals.

**Conclusions/Significance:**

Across therapeutic areas, biomedical research funding increased substantially, appears aligned with disease burden in high income countries, but is not linked to new drug approvals. The translational gap between funding and new therapies is affecting all of medicine, and remedies must include changes beyond additional financial investment.

## Introduction

Biomedical research in the United States has been the beneficiary of investment by many public and private sources. This investment reflects its importance to society, whether measured by human suffering and the burden of disease or by commercial and economic terms. Current total annual funding for biomedical research in the U.S. is approximately $100 billion, and over the past decade has tripled in nominal dollars and doubled after adjusting for inflation [1], [2]. However, the rise in funding has not been mirrored by an increase in new therapies [2]. Within each therapeutic area in medicine (e.g., oncology, cardiology), the sources of funds, their relationship to disease burden, and the number of recently developed therapies is generally not known. Therefore, we sought to (1) estimate U.S. funding by therapeutic area, (2) determine whether this funding is aligned with disease burden, and (3) evaluate whether this investment has translated into therapeutic advances. Comparing the productivity of biomedical research across therapeutic areas will help guide and inform private investments and public research policy [3], [4].

## Methods

### Therapeutic Areas Examined

We selected based on available data and defined nine therapeutic areas within medicine (cardiovascular, endocrine, gastrointestinal, genitourinary, HIV/AIDS, infectious disease excluding HIV, neuroscience, oncology, and respiratory) based on U.S. Bureau of Census Industrial Report product codes for pharmaceutical preparations, except biologicals [5]. For neuroscience, we used previously published data [6] but revised the National Institutes of Health (NIH) funding estimates to exclude HIV/AIDS research. With the exception of HIV/AIDS, each therapeutic area was broad and included multiple medical conditions. For example, funding for neuroscience included funding directed at neurological disorders (e.g., stroke, Parkinson disease), mental health (e.g., depression, schizophrenia), substance abuse, and sensory organs besides skin.

### Biomedical Research Funding by Therapeutic Area

#### National Institutes of Health

We allocated NIH funding by assigning each Institute's annual appropriation to a therapeutic area (e.g. appropriations for the National Cancer Institute were assigned to oncology) [7]. We allocated appropriations for Institutes that covered multiple therapeutic areas based on funding for disease divisions within each Institute, as outlined in each Institute's Congressional Budget Justification.

We quantified HIV/AIDS research support from Office of AIDS Research Congressional Budget Justifications (1998–2005) [8] and its budget office (1995–1997) (Wendy Wertheimer, Office of AIDS Research Information Dissemination, historical data, 2007). We estimated infectious disease research funding excluding HIV using appropriations for the National Institute of Allergy and Infectious Disease and the John E. Fogarty International Center. Cardiovascular research funding was estimated using monies directed to “heart and vascular research” and “blood diseases and resources” by the National Heart, Lung, and Blood Institute (NHLBI) [9]. Respiratory research funding was estimated by combining funding for “lung diseases” and “sleep disorders.” The ratio of cardiovascular to respiratory NHLBI research funding was applied to the balance of NHLBI appropriations for each year to distribute proportionately all remaining funding between cardiovascular and respiratory research. Endocrine, gastrointestinal, and genitourinary research funding were identified through appropriations to the National Institute of Diabetes and Digestive and Kidney Diseases (NIDDK) [10] and its budget office (Chris Porter, NIDDK Office of Financial Management and Analysis, historical data, 2007).

Using this methodology, we categorized $8.1 (72%) of $11.3 billion in total NIH appropriations in 1995, and $20.2 (72%) of $28 billion in total NIH appropriations in 2005. The remaining research funding included appropriations to Institutes and Centers without a clear link to a therapeutic area examined.

#### Pharmaceutical firms

We estimated domestic biomedical research funding from pharmaceutical firms using funding data from the Pharmaceutical Research and Manufacturers of America (PhRMA) for 1995–2000 [11]–[13] and from Thomson CenterWatch thereafter [14]. PhRMA reports domestic research and development expenditures stratified by U.S. Bureau of Census Industrial Report product codes [5]. Cardiovascular and respiratory research support were identified directly. The remaining product codes were separated into more focused therapeutic areas based on the value of shipments of pharmaceutical preparations within each product code [5].

Because Thomson CenterWatch estimates included foreign research and development expenditures, they were greater than PhRMA's totals (P. Dewberry, personal communication, September 6, 2006). To account for this disparity we conservatively decreased Thomson CenterWatch estimates to the total domestic research and development expenditures reported by PhRMA [15], keeping the proportions across therapeutic areas constant. Data for years 2001, 2003, and 2005, which were not reported by Thomson CenterWatch, were interpolated linearly from neighboring years. Thomson CenterWatch research expenditure estimates were stratified into the same six product codes as the PhRMA funding data, and the same methodology was used to allocate funds into the nine therapeutic areas. Missing data (5 of 99 total cells) was estimated by linear interpolation from neighboring years.

#### Biotechnology and medical device firms

We estimated research and development expenditures by the 10 largest US-based biotechnology and medical device firms that were not members of PhRMA in 2005. We selected the top 10 biotechnology companies (Genentech, Gilead Sciences, Biogen Idec, MedImmune, Celgene, ImClone, Medicis, ViroPharma, Protein Design Labs, and Intermune) top 10 medical device firms (Medtronic, Baxter, Tyco International, Boston Scientific, Becton Dickinson, Stryker, Guidant, Zimmer, St Jude Medical, and Biomet) based on their 2005 revenues using Standard Industry Classification codes [16] and each firm's financial reports [17].

We obtained research and development expenditures from each firm's Security and Exchange Commission filings [17] and used several methods to estimate expenditures for each therapeutic area. We first assigned each of a firm's approved products to a therapeutic area based on the product's primary therapeutic indication. When a firm's financial report clearly defined its research and development expenditures by product, we assigned expenditures to therapeutic areas based on the product's primary therapeutic indication (n = 3 firms). If research and development expenditures were not listed by product, we estimated each product's portion of research and development spending by taking the ratio of product-specific revenues to total revenues and applying this to total research and development expenditures (n = 12). If neither research and development expenditures nor total revenues were listed by product (n = 5), we then took the product-specific proportion of revenue from the earliest year available and applied this proportion to total research and development spending for all previous years.

#### Not-for-profit organizations

Not-for-profit organizations included foundations and voluntary health organizations. We included the ten largest foundations that funded medical research for each year 1998–2005 (total of 28 foundations over the course of 8 years), as identified by Foundation Center [18], and ten voluntary health organizations with the largest research expenditures in 2004, as identified by Research!America [19]. We then examined research support from annual reports (if available) or by contacting organizations directly. Of the 38 total not-for-profit organizations, 16 did not support research that could be specifically assigned to therapeutic areas defined in this study and 5 did not have data available (including the largest foundation). Of the 17 remaining organizations, 11 had data for all years and 6 had data available for only several years (mean of 3 years). Research funding for each organization was allocated to a therapeutic area based on information provided by annual reports [20]–[36].

### Disease Burden

We obtained projected disease burden estimates, as measured in disability-adjusted life years (DALYs), for both the world and high-income countries from the World Health Organization Global Burden of Disease report for 2015 [37], [38]. WHO disease categories were more focused than those in the U.S. Census Bureau Industrial Report, and thus several categories (e.g., “neuropsychiatric conditions” and “sense organ diseases”) were combined to match the defined therapeutic areas used here (e.g., neuroscience).

### Therapeutic Outputs

We obtained the number of approved drugs directly from the U.S. Food and Drug Administration (FDA) website [39]. We assigned a therapeutic area to each new drug application approval from 1995 through 2005, based on the U.S. Bureau of Census pharmaceutical product codes [5], [40]–[42]. We tabulated all new drug application and all new molecular entities [40]–[42] approvals for each of the therapeutic areas.

### Statistical Analysis

We used the Biomedical Research and Development Price Index to adjust spending to 2005 dollars [43]. To determine whether funding in 2005 was aligned with disease burden, we used SAS version 9.1 to calculate Spearman rank correlations between total funding and disease burden for 2015 across therapeutic areas. We used projected DALYs for 2015 as the primary metric because research investments may take up to a decade or more to be realized in the form of new treatments. Rank correlations between 2015 disease burden and major funding sources (NIH or industry) were also calculated. To evaluate whether funding was associated with FDA approvals, we calculated rank correlations between total funding (for all therapeutic areas) in each year (e.g., 1995 funding) and FDA approvals eight years hence (e.g., 2003 approvals), which approximates the time to develop a drug once it enters clinical testing [44]. Because an eight-year lag period is insufficient to account for pre-clinical development where the majority of NIH funding is directed [2], we also calculated rank correlations between FDA approvals and industry funding alone (pharmaceutical, biotechnology, and medical device firms). The a priori level of significance used for correlations was 0.05 (without adjusting for multiple comparisons).

## Results

After adjusting for inflation, financial support for domestic biomedical research from 1995 to 2005 increased between 43% and 369% in the therapeutic areas examined (Table 1). Neuroscience, oncology, and cardiovascular research received the largest total funding and accounted for 57% of 2005 total research expenditures in this analysis. Gastrointestinal and genitourinary research funding experienced the greatest increase.

**Table 1:** Funding for Biomedical Research by Therapeutic Area, 1995–2005.

	US $ in Billions												
	1995	1996	1997	1998	1999	2000	2001	2002	2003	2004	2005	Percent change	Inflation-adjusted percent change*
Neuroscience	4.8	5.5	6.5	7.6	7.3	7.7	9.0	10.2	11.3	12.6	13.6	184%	101%
Oncology	3.9	3.9	4.1	4.6	5.5	5.3	6.5	7.0	7.5	8.2	8.8	126%	60%
Cardiovascular	3.8	3.9	4.5	5.0	5.0	5.0	6.0	6.3	6.9	7.6	8.4	120%	56%
Endocrine	1.5	1.9	2.0	2.2	2.4	2.9	3.8	4.4	4.8	5.2	5.8	285%	172%
Infectious disease (excluding HIV)	2.0	2.2	3.1	3.1	2.1	2.7	3.2	3.5	4.5	5.1	5.4	168%	89%
HIV/AIDS	1.7	1.8	2.2	2.8	3.1	3.2	3.6	3.4	4.0	4.5	5.0	196%	110%
Gastrointestinal	0.4	0.6	0.6	0.7	0.8	0.9	2.0	3.3	3.8	3.4	2.9	562%	369%
Respiratory	1.1	1.3	1.3	1.1	1.3	1.2	1.6	1.7	1.8	2.1	2.2	102%	43%
Genitourinary	0.3	0.3	0.4	0.5	0.6	0.7	1.6	2.4	1.7	2.0	1.7	443%	284%
Total	19.5	21.4	24.8	27.5	28.0	29.6	37.3	42.1	46.4	50.7	53.8	175%	95%
Adjusted total^a^	27.6	29.5	33.2	35.7	35.3	36.0	43.7	47.8	50.7	53.4	53.8		

* Adjusted for inflation by the Biomedical Research and Development Price Index.

**Figure 1:**
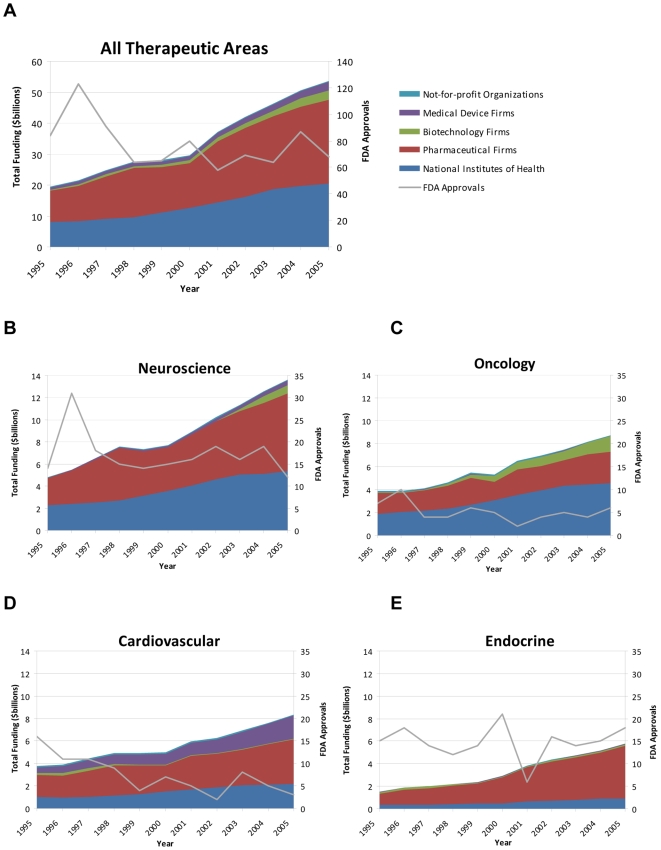
Research support, sponsors, and FDA new drug approvals from 1995 – 2005 for a) all therapeutic areas, b) neuroscience research, c) oncology research, d) cardiovascular research, and e) endocrine research.

**Figure 2:**
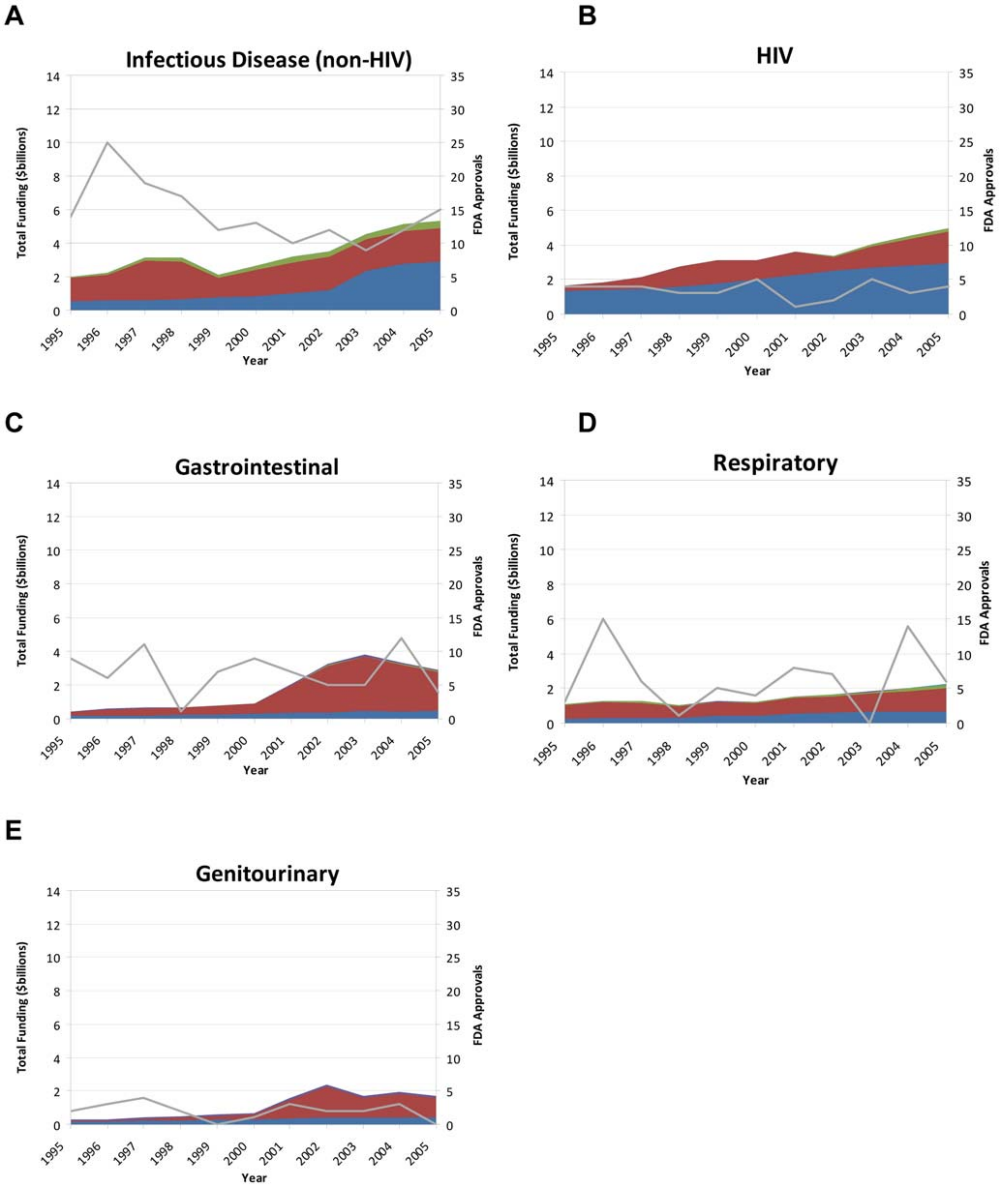
Research support, sponsors, and FDA new drug approvals from 1995 – 2005 for a) infectious disease (non-HIV) research, b) HIV research, c) gastrointestinal research, d) respiratory research, and e) genitourinary research.

The pharmaceutical industry was the largest sponsor of research across most therapeutic areas, ranging from 32% of oncology research support to 80% of endocrine research in 2005 (Figures 1a–e and Figures 2a–e). However, NIH provided the majority of support for HIV/AIDS (59%), infectious disease (excluding HIV) (54%), and oncology (52%) research. Oncology (42%) was the largest recipient of biotechnology funding, while 73% of medical device funding targeted cardiovascular disease.

**Figure 3:**
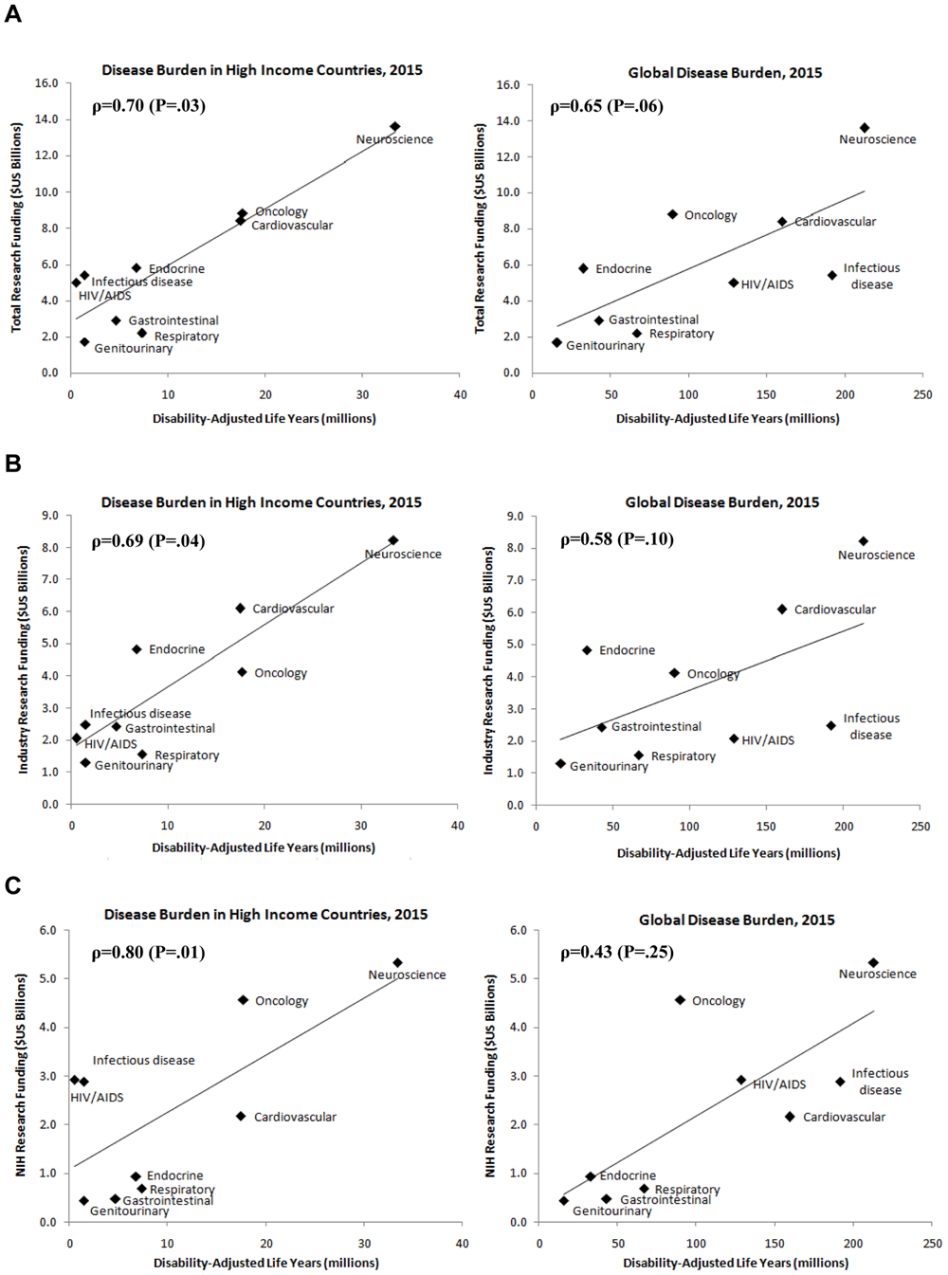
Correlation between a) total, b) industry, and c) NIH research funding in 2005 and projected disease burden in 2015 for high income countries and the world.

Total funding in 2005 was correlated with projected 2015 disease burden in high income countries (ρ = 0.70; P = 0.03) and likely correlated with 2015 global disease burden, but the latter did not reach traditional standards of statistical significance (ρ = 0.65; P = 0.06) (Figure 3a). Industry funding in 2005 was more strongly aligned with 2015 disease burden in high income countries (ρ = 0.69, P = 0.04) than with global disease burden projections (ρ = 0.58; P = 0.10) (Figure 3b). Conversely, NIH funding was more strongly aligned with 2015 global disease burden (ρ = 0.80; P = 0.01) than with disease burden in high income countries (ρ = 0.43; P = 0.25) (Figure 3c).

Across therapeutic areas, the number of total new approvals from 1995 to 2005 generally was flat (Figures 1a–e, Figures 2a–e, and Table 2). The number of approvals for new molecular entities also did not increase substantially for any therapeutic area during this time period (Table 3), and overall, approvals decline by 46%.

**Table 2:** Total Number of Drugs Approved by US Food and Drug Administration by Therapeutic Area, 1995–2005.

	1995	1996	1997	1998	1999	2000	2001	2002	2003	2004	2005
Neuroscience	14	31	18	15	14	15	16	19	16	19	12
Oncology	7	10	4	4	6	5	2	4	5	4	6
Cardiovascular	16	11	11	9	4	7	5	2	8	5	3
Endocrine	15	18	14	12	14	21	6	16	14	15	18
Infectious disease	14	25	19	17	12	13	10	12	9	12	15
HIV	4	4	4	3	3	5	1	2	5	3	4
Gastrointestinal	9	6	11	1	7	9	7	5	5	12	4
Respiratory	3	15	6	1	5	4	8	7	0	14	6
Genitourinary	2	3	4	2	0	1	3	2	2	3	0
**Total**	**84**	**123**	**91**	**64**	**65**	**80**	**58**	**69**	**64**	**87**	**68**

**Table 3:** New Molecular Entities Approved by US Food and Drug Administration by Therapeutic Area, 1995–2005.

	1995	1996	1997	1998	1999	2000	2001	2002	2003	2004	2005
Neuroscience	4	9	7	6	6	6	5	4	1	6	1
Oncology	3	6	3	2	4	3	1	2	3	4	3
Cardiovascular	9	4	7	7	2	3	3	2	2	1	0
Endocrine	3	5	6	1	3	5	3	0	3	2	6
Infectious disease	2	7	4	2	5	2	3	3	3	3	3
HIV	2	3	3	2	1	1	1	0	3	0	1
Gastrointestinal	2	1	2	0	2	4	0	3	3	1	0
Respiratory	1	4	0	1	2	0	2	0	0	1	0
Genitourinary	0	2	1	1	0	0	2	1	1	3	0
**Total**	**26**	**41**	**33**	**22**	**25**	**24**	**20**	**15**	**19**	**21**	**14**

Higher total research funding was not associated with a higher number of FDA approvals. Neither total (ρ = 0.31; P = 0.42) nor industry funding (ρ = 0.48; P = 0.19) was correlated with total approvals over a lag period of eight years.

## Discussion

Like biomedical research as a whole, research funding within the therapeutic areas examined adjusted for inflation doubled over the past decade. The principal source (industry or NIH) of funding varied by therapeutic area and research funding appeared to be aligned with measures of disease burden. However, across therapeutic areas, the increase in funding has not been accompanied by an increase in the number of new therapies available. Therefore, other factors must account for the mismatch between investment and output, at least in drug development.

While industry funds the majority of research as a whole [2], [19], at least three therapeutic areas – HIV/AIDS, infectious disease excluding HIV, and oncology – received the majority of their funding from the NIH. This finding reflects economic externalities (influences, benefits or harms to third parties) that are not reflected in markets [45]. These accrue in HIV/AIDS and other infectious diseases, where research investment reflects concern for public health. Political factors also influence public financing for these conditions, as HIV and cancer have high visibility [46].

Biomedical research funding appeared to be aligned with disease burden, at least in high-income countries. Previous research has also found NIH funding to be correlated with disease burden [3]. Unlike NIH funding, U.S. industry funding was associated with disease burden in high-income countries but not with global measures. The principal difference between NIH and industry appears to be the differential funding of HIV/AIDS and infectious diseases. While industry funding for infectious diseases and HIV/AIDS is proportional to disease burden in high-income countries, industry research funding is not on par with the global burden of HIV/AIDS and other infectious diseases. This discrepancy highlights the importance of public payers and foundations, such as the Bill & Melinda Gates Foundation [47], the Rockefeller Foundation [48], and others, whose funding priorities reach beyond the U.S. boundaries.

While funding was correlated with disease burden, it was not associated with an increase in new therapies, even when incorporating a lag of eight years between funding and new drug approvals. Probable explanations are longer and more complex clinical trials and the associated additional cost of drug development, with current estimates ranging from $600 million to $1.2 billion [44], [49]. Likewise, the introduction of the development of large molecule biopharmaceuticals may have increased the cost of drug development, but research suggests that the costs of developing a biopharmaceutical are comparable to those compounds produced by traditional pharmaceutical firms [50]. New basic scientific knowledge also requires time before it is ready for pre-clinical or clinical testing. Therefore, future therapeutic advances may be forthcoming, and one study of the drug development pipeline for Parkinson disease suggests that the pipeline of available therapies is increasing [51]. However, the declining trend in the number of new molecular entities is worsening rather than improving. In 2007, the FDA approved 19 new drugs, the fewest in 24 years [52]. The increased funding and absent rise of new drugs in all therapeutic areas adds to the growing concerns about the productivity of biomedical research [2], [6], [53], [54] and underscore a need to examine non-economic factors in the search for new treatments.

Economists [55] and historians of science [56] stress that financial investment is but one of several elements that are necessary for scientific progress. Other requirements include the ready supply of talent (with relevant skill), favorable geography (proximity of universities and companies), and a culture that supports mobility between institutions (of people, ideas, and material). These factors have been recognized by the NIH, foundations, and companies as priorities equal to additional investment [57] and non-financial factors, such as exploring partnerships between academia and industry, are receiving growing attention in the scientific press [57], [58]. The challenge is to increase *productivity*, which is not commonly the focus of scientists. Money, while necessary, is not sufficient to find new drugs. Possible solutions to improving research productivity have been offered and include decreasing the costs of clinical trials [59], modifying the economic incentives that pharmaceutical companies face (to favor high impact/high cost conditions) [60], increasing the scale of research, open dissemination of negative results, and reorganizing research enterprises to bring talent, instrumentation, information, and material together in new ways [58], [61], [62]. Importantly, as demonstrated by recent experience, further increases in the investment in research are probably inadequate, unless they are complemented by such non-financial factors.

Our study focused on a narrow measure of output, new drug approvals. While this metric is important and highly relevant to the pharmaceutical industry, which funds most U.S. biomedical research, other metrics may be more important to public health, the economy, and science. For example, life expectancy in the U.S. continues to increase, including for the decade 1990–2000 [63]. One large factor driving this increase is the reduction in cardiovascular deaths with at least half this reduction attributable to medical advances [63]. Economic research suggests that societal gains from investment in research have been enormous and advances that result in even modest reductions in cancer mortality would more than justify substantial additional investments in biomedical research [64]. In addition to the historical medical advances, an indicator of more recent scientific advances is patent activity. Research shows that the number of patents granted to U.S. medical school faculty increased dramatically from 1981 to 2000 [65]. While the increase in patents and the economic potential of additional therapeutic advances are promising, the challenge is realizing this potential.

Our financial analysis was also limited by its geographic scope and the data available. Our analysis was limited to the U.S. Funding for biomedical research outside the U.S. is growing. For example, PhRMA estimates that in 2008 research and development abroad was $11.8 billion or 24% of total research and development for its member companies. By comparison, in 2005 funding abroad was $3.3 billion and 22% of total [66]. Evaluating global biomedical research funding, its trends, and relationship to disease burden remain open areas for investigation. We were able to tally funding from the two largest sponsors of U.S. biomedical research that was directed toward one of the selected therapeutic areas (over 84% of pharmaceutical industry funding and 70% of NIH funding). However, not all funding is from one of the principal sponsors and not all is directed toward a specific therapeutic area. Consequently, we were able to account for approximately half of all U.S. biomedical research funding ($46.4 billion of an estimated $94.3 billion in 2003) [2]. For the $48 billion that was outside the scope of this investigation, $27 billion was from industry (principally biotechnology and medical device industry) and could not be categorized by therapeutic area, $13 billion was from sources not included in this analysis (e.g., federal sources outside the NIH, state and local governments, private funds), $7 billion was from the NIH that is not targeted at a particular therapeutic area (e.g., National Library of Medicine, National Human Genome Research Institute), and approximately $1 billion targeted therapeutic areas outside the scope of this paper (e.g., dermatology). In addition, the data gathered were from different sources that used various methods in their compilation. However, the data do facilitate a comparison of funding across therapeutic areas and an analysis of funding trends. Our funding data was based on a convenience sample; therefore, our inferential testing may be biased. For example, individuals who fund research were not included in our analysis, and their funding priorities may differ from those of industry or government.

With the exception of HIV/AIDS, we could not allocate funding to a single disease. Thus the analyses, including correlations between financing and disease burden and financing and new drugs, performed are necessarily imperfect. In evaluating disease burden, we relied on estimates from the World Health Organization for DALYs as opposed to quality-adjusted life years, which are frequently used in pharmacoeconomic analyses.

In conclusion, funding from the major U.S. research funders across therapeutic areas in medicine has increased over the last decade, is aligned with disease burden in high income countries, but is not linked to the development of new medical therapies. Our findings suggest that translating investment in biomedical research into new therapies is becoming increasingly difficult across all areas of medicine. Identifying means to either decrease the cost of research or increase the output of new therapies will challenge all those who invest in research, and these challenges will only grow as funding constraints from industry [52] and governments [67] become more apparent.

## Acknowledgments

We thank P. Dewberry from Thomson CenterWatch, Chris Porter and the National Institute of Diabetes and Digestive and Kidney Diseases, Wendy J. Wertheimer and the Office of AIDS Research of the National Institutes of Health, the National Institute of Arthritis and Musculoskeletal and Skin Diseases budget office, and the W.M. Keck Foundation for assistance in providing data.
